# Is Human Immunodeficiency Virus Infection a Risk Factor for *Strongyloides stercoralis* Hyperinfection and Dissemination

**DOI:** 10.1371/journal.pntd.0001581

**Published:** 2012-07-31

**Authors:** Marc O. Siegel, Gary L. Simon

**Affiliations:** Division of Infectious Diseases, George Washington University Medical Center, Washington, DC, United States of America; The George Washington University Medical Center, United States of America

*Strongyloides stercoralis* was first identified in post-mortem examination of the gastrointestinal tract of five French soldiers from Cochin, China, in 1876. Since then it has been recognized that infection with this organism can persist for decades. This is due to an autoinfective process whereby rhabditiform larvae that are excreted by the adult worm are converted to infectious filariform larvae in the large intestine where they can then reinfect the host. Under normal conditions this conversion in humans is quite limited, with most conversions occurring in the soil. Occasionally large numbers of rhabditiform larvae transform into infective filariform larvae in the human gastrointestinal tract, which results in a more severe form of the autoinfective cycle. This is referred to as hyperinfection syndrome and can result in dissemination of the larvae to other organs in the host with potentially fatal consequences.

The hyperinfection syndrome has been linked to immunosuppression. In particular, conditions that impact on cell-mediated immunity have been more closely identified as risk factors for the development of this syndrome. The two conditions that have been most frequently recognized as predisposing factors for the development of the hyperinfection syndrome are corticosteroid use [1], [2] and human T-lymphotropic virus type 1 (HTLV-1) infection [3]. Studies of disseminated disease in organ transplant patients, asthmatics, patients with chronic lung disease, and patients with autoimmune disease have shown that corticosteroid therapy has been a common denominator in the development of severe infection.

When acquired immune deficiency syndrome (AIDS) was first described, it was predicted that there would be an outbreak of disseminated strongyloidiasis, especially in patients from the developing world, where *S. stercoralis* is endemic. Prior to the recognition of the human immunodeficiency virus (HIV), the diagnosis of AIDS was based on the presence of a selected group of opportunistic infections in patients who had no other identifiable predisposing condition. Disseminated strongyloidiasis was among this list of opportunistic infections. These initial concerns do not appear to have been warranted. In necropsy studies in HIV-infected patients in both Brazil and Africa, areas where the incidence of strongyloidiasis is high, there was not a single case of disseminated disease [4], [5]. Although it is possible that cases might have gone unrecognized, since clinical monitoring in those parts of the world where strongyloidiasis is endemic might have been less comprehensive than in more developed areas, the relative paucity of cases led the CDC and the WHO to remove disseminated strongyloidiasis from its list of signature infections in 1987. Since then there have been only 40 cases of hyperinfection and disseminated strongyloidiasis in HIV-infected individuals reported in the medical literature. Most of these individuals had AIDS and many were also receiving corticosteroids.

Of note, HIV infection does not protect individuals from acquiring intestinal strongyloidiasis. Several studies have documented increased rates of *S. stercoralis* infection among HIV-infected individuals. Assefa et al. found a 21-fold increased prevalence of *S. stercoralis* infection among HIV-positive compared to HIV-negative patients in southern Ethiopia [6]. Studies in Brazil have shown similar results [7]. However, this increased predilection for intestinal *S. stercoralis* infection among HIV-infected individuals does not seem to be predictive of an increased incidence of hyperinfection and dissemination.

The predominant immunosuppressive effect of HIV infection is a cellular immune deficiency as evidenced by a progressive decline in CD4+ lymphocytes. However, within the CD4+ cell population, there is a relatively greater decline in the activity of the type 1 T helper (Th1) cells than the type 2 T helper (Th2) cells [8], [9]. Th1 cells produce a variety of pro-inflammatory cytokines that modulate the cellular immune response including interferon gamma (IFN-γ), interleukin 2 (IL-2), and tumor necrosis factor alpha (TNF-α) [10]. Th2 cells, on the other hand, are more active in mediating humoral immunity and produce cytokines IL-4, IL-5, IL-10, and IL-13. The concept of a Th1 to Th2 cytokine shift in the course of HIV infection has been advanced by some investigators as a marker for HIV progression [10]. Others have not confirmed these findings and they remain somewhat controversial [11]. Nevertheless, whereas there is profound loss of Th1 immune activity in patients with advanced AIDS, there may be little change in Th2-mediated cytokine activity. Indeed, levels of the Th2 cytokines IL-4 and IL-10 have been found to be higher in HIV-infected patients, both with and without opportunistic infections, than in uninfected controls [12]. The potential consequence of this is that for many co-infected patients, the Th2-mediated response to helminthic infections may be conserved.

In general, the Th2 immune response is dominant in patients with helminthic infections. IL-4 and IL-5 stimulate IgE production, which in turn causes mast cells to degranulate and goblet cells to secrete mucous [13]. The mucous facilitates trapping and expulsion of the helminths, while mast cells prevent attachment and invasion of the worms to the intestinal wall and promote peristalsis to aid in the expulsion of the parasites [14]. IL-5 also stimulates the production, migration, and activation of eosinophils [14].

Another Th2-mediated cytokine, IL-5, acts as an eosinophil colony stimulating factor. Eosinophils play a major role in host defense against helminthic infections. Killing of parasites is due to the release of eosinophilic cytoplasmic granules on the surface of the eosinophils. Eosinophils are directly involved not only in the innate immune response to the helminthic larvae, but also in eliciting an adaptive immune response. Padigel et al. demonstrated that eosinophils act as antigen-presenting cells when exposed to *S. stercoralis* antigens, thereby stimulating antigen-specific Th2 cytokine production [15].

Elevated IgE levels are commonly found in patients with helminthic infections, and type-specific anti-*Strongyloides* IgE antibody has been demonstrated in patients with *S. stercoralis* infection [16]. HIV infection is also associated with high IgE levels, with higher levels found in patients with more advanced infection; that is, those with lower CD4+ cell counts [17]. There is evidence to suggest that HIV infection promotes the production of IL-4 and IL-13, B-cell growth factors. HIV-1 glycoprotein 120 (gp120) is a potent stimulus for release of these cytokines through an interaction with the V_H_3 region of IgE that is bound to the FcεRI region on basophils and mast cells [18]. In fact, it has been suggested that HIV gp120 acts as an allergen promoting increased production of IgE [19]. Whether the elevated IgE levels found in co-infected patients are type-specific and whether this is a key factor in preventing dissemination have not been demonstrated, but this is an area for further investigation.

Helminthic infections themselves promote immune activation leading to immune dysregulation and result in a decrease in CD4+ lymphocytes and an increase in CD8+ cells [20], [21]. However, the observed loss in CD4+ cells in patients with helminthic infections does not reach the level seen in HIV infection. The role of helminthic induced immune activation and its relationship to the development of vaccines against *S. stercoralis* and other helminths is a potential fertile area for further research.

In contrast to the relatively preserved Th2 activity associated with HIV infection, individuals infected with HTLV-1, a retrovirus that has been associated with an increased risk of disseminated strongyloidiasis, have an immunologic shift to a Th1 cell type response. HTLV-1-infected lymphocytes produce increased levels of IFN-γ and reduced levels of IL-4 and IL-5 [22]. Mitogen-stimulated PBMCs from HTLV-1-infected patients infected with *S. stercoralis* produce higher levels of IFN-γ and lower levels of IL-4 than control patients [23]. In these patients total serum IgE levels were inversely correlated with mitogen-stimulated IFN-γ production in these patients. *S. stercoralis*-specific IgE levels were also reduced, but the correlation did not reach statistical significance.

Treatment of HIV infection is often associated with improvement in the host non-specific inflammatory response. There have been only five cases of immune reconstitution inflammatory syndrome (IRIS) reported in patients with *S. stercoralis* infection [24]. Three of these patients had a syndrome that was consistent with disseminated infection, but two of these patients had received courses of corticosteroids prior to their diagnosis, making it unclear whether their infection was precipitated by the IRIS.

There are a number of unanswered questions regarding this topic. The first is that the underlying presumption that HIV does not lead to disseminated infection is based on the absence of data as well as the WHO and CDC list of signature infections that have been associated with HIV. Proof that something does not exist is difficult, but until such data are available, the current epidemiology does suggest that HIV is not a risk factor for disseminated strongyloidiasis. The impact of HIV on the susceptibility to infection with *S. stercoralis* is not well-defined. Does chronic immune activation make one more susceptible to *S. stercoralis*? What is the relative role of IgE antibodies, eosinophils, and/or other Th2-mediated activity in preventing dissemination? Further study on the immune interaction between HIV, *S. stercoralis*, and other helminths may provide useful insight into the development of helminthic vaccines.

In summary, despite the fact that HIV and glucocorticosteroids are viewed as similar cell-mediated immune suppressants, their immunosuppressive activities are quite different. HIV infection results, primarily, in a loss of Th1 activity. In comparison, Th2 activity in the HIV-infected individual may be impaired to a much lesser degree, or may even be augmented. This does not necessarily indicate that infection with HIV actually leads to enhanced activity specifically against *S. stercoralis* or even that the HIV-infected host has the equivalent anti-*Strongyloides* activity as an immunologically normal individual. However, it may be the Th2 activity in the HIV host helps to prevent dissemination of *S. stercoralis* in the HIV-infected population.
